# Interleukin-1 beta converting enzyme is necessary for development of depression-like behavior following intracerebroventricular administration of lipopolysaccharide to mice

**DOI:** 10.1186/1742-2094-10-54

**Published:** 2013-05-01

**Authors:** Marcus A Lawson, Robert H McCusker, Keith W Kelley

**Affiliations:** 1 Neuroscience Program, University of Illinois at Urbana-Champaign, Urbana, IL, USA; 2 Integrative Immunology and Behavior Program, Department of Animal Sciences, University of Illinois at Urbana-Champaign, Urbana, IL, USA; 3 Department of Pathology, Colleges of ACES and Medicine, University of Illinois at Urbana-Champaign, Urbana, IL, USA

## Abstract

**Background:**

Interleukin-1 beta converting enzyme (ICE, caspase 1) is a cysteine protease that processes immature pro-IL-1β into active mature IL-1β. IL-1β is a pro-inflammatory cytokine that mediates many of the physiological and behavioral responses to inflammation. Genetic deletion of ICE has previously been shown to prevent some negative physiologic responses to lipopolysaccharide (LPS)-induced inflammation.

**Methods:**

Here we used a preclinical murine model to test the hypothesis that ICE is necessary for development of depression-like behaviors following intracerebroventricular (ICV) treatment with LPS. Adult male ICE knockout (ICE KO) and congenic wild-type C57BL/6 J (WT) mice were administered LPS either ICV at 100 ng/mouse or intraperitoneally (IP) at 830 μg/kg body weight or an equal volume of saline as controls. Mice were monitored up to 48 h after treatment for both sickness and depression-like behaviors.

**Results:**

LPS given ICV induced a loss of body weight in both WT and ICE KO mice. This sickness response was similar between WT and ICE KO mice. As expected, LPS administered ICV increased immobility in the forced swim test (FST) and decreased sucrose preference in WT mice but no change in either of these two depression-like behaviors was observed in ICE KO mice. Expression of TNF-α and CD11b in brain was lower in ICE-KO mice at 24 h following ICV administration of LPS compared to WT mice. In contrast, when LPS was given systemically, sickness response, depression-like behaviors, and expression of these genes were similar between the two strains of mice.

**Conclusions:**

These findings indicate that ICE plays a specific role in depression-like behavior induced by a central inflammatory stimuli even though it is not required when LPS is administered systemically.

## Background

During the past two decades, substantial support has accumulated for the idea that the co-morbid symptoms associated with clinical depression and neurodegenerative diseases share inflammation as a common an important component of their etiology [1-3]. Moreover, prevalence rates for neuropsychiatric disorders along with chronic inflammatory diseases, such as type II diabetes and heart disease within the general population, are rapidly increasing [4,5]. Similarly, the incidence of neurodegenerative diseases such as Alzheimer’s and Parkinson’s is rising, predominately due to increasing life-span [6,7]. As a result of these challenges, therapies targeted to alleviate inflammation are now viewed as potential treatments for clinical depression [8].

Preclinical research using animals to model human depression has provided significant insight into which inflammatory mediators may be good therapeutic drug targets [9-12]. The overall goal is to diminish inflammation by blocking the production of inflammatory cytokines such as IL-1β [13,14]. IL-1β plays a prominent role in neurodegenerative processes [9,15] and has recently been identified for its role in murine models of depression-like behavior [10,16]. In agreement with these preclinical models, evidence from human studies indicates higher cerebral spinal fluid IL-1β levels in patients suffering from acute depression [17].

Systemic administration of lipopolysaccharide (LPS) induces expression and activity of interleukin-1ß (IL-1β) and IL-1β-converting enzyme (ICE) in myeloid-derived cells that are resident to many organs, including brain microglia [18,19]. ICE is the primary enzyme responsible for cleavage of pro-IL-1β and pro-IL18 to fully processed mature cytokines, eventually leading to increased secretion of active IL-β and IL-18. ICE activation is linked to assembly and activation of the inflammasome following recognition of numerous pathogen- or danger-associated molecular patterns and toll-like receptor binding [20-22]. ICE activity has been shown to influence food intake during inflammation [23,24], presumably through its role in processing IL-1β. Evidence from experiments utilizing ICE KO mice demonstrated that these mice have impaired processing of pro-IL-1ß and reduced secretion of IL-1β following stimulation with LPS [19]. IL-1β plays a prominent role in brain during inflammation as first evidenced by experiments that demonstrated IL-1β-induced activation of hypothalamic-pituitary-adrenal axis and stress responses [25-27]. Further, ongoing research continues to highlight the influence of IL-1β in many chronic inflammatory diseases and mood disorders. Thus targeting of ICE represents a possible alternative therapeutic route to target IL-1β.

We examined the role of ICE in depression-like behaviors utilizing murine models of systemic and central inflammation following an IP (systemic) or ICV (central) injection of LPS, respectively. Based on our previously published results [23,24], we hypothesized that ICE KO mice would be protected from depression-like behavior induced by centrally administered LPS but not from depression-like behaviors induced by systemic LPS. Here we report that ICE KO mice were protected from central inflammation-induced depression-like behavior as measured by two well-accepted behavioral tests [28]. However, when challenged with systemic LPS, ICE KO mice displayed depression-like behaviors comparable to WT mice. This disparate behavioral response corresponds to decreased brain expression of pro-inflammatory cytokines and markers of glial activation in ICE KO mice following ICV but not IP LPS. Consequently, therapies designed to inhibit ICE activity may be a viable treatment of comorbid depression associated with inflammatory diseases of the central nervous system.

## Methods

### Animals

C57BL/6 J (WT) mice were purchased from Jackson Laboratories (Stock #000664, Bar Harbor, ME, USA). ICE KO mice on a C57BL background were kindly provided by Dr. Richard Flavell (Yale University School of Medicine [29]). These mice are genetically identical to mice now also available from Jackson Laboratories (Stock #016621). In these experiments, WT and ICE KO male mice were individually housed and provided *ad libitum* access to Teklad 8640 chow and water in a temperature- (23°C) and humidity- (45%) controlled room and maintained on a 12:12 hour light:dark cycle (lights off at 10:00). Mice were acclimated to these conditions for at least 2 weeks prior to initiation of any procedure. When mice reached at least 10 weeks of age, those mice to be treated ICV were surgically implanted with a guide cannula (Plastics One, Roanoke, VA, USA) directed toward the lateral ventricle as previously described [30]. The coordinates for implantation were determined utilizing *The Mouse Brain in Stereotaxic Coordinates*[31] and cannulas were placed at 1.5 mm lateral, 0.6 mm posterior, and 1.3 mm dorsal with respect to bregma. These coordinates placed the guide cannula 1 mm dorsal to the lateral ventricle. Mice were allowed to recover from surgery for 10 to 14 days before being treated. Prior to any treatment, mice were handled daily to habituate them to being restrained and manipulated. All procedures performed on the mice were in compliance with the National Institutes of Health guidelines and approved by the University of Illinois at Urbana Champaign Institutional Animal Care and Use Committee.

### Treatments

Body weights were measured on the day of treatment prior to injections and 24 h later to evaluate sickness response. Mice were injected using a single internal injector cannula for mice (Plastics One, Roanoke, VA, USA) which extended 1 mm beyond the tip of the guide cannula to reach the lateral ventricle. Mice were injected at the onset of the dark cycle using a 10 μL gas-tight syringe (SGE Incorporated, Austin, TX, USA) to administer 1 μL of endotoxin free phosphate buffered saline (PBS) or 100 ng LPS (from *Escherichia coli* O127:B8, Sigma Aldrich, St Louis, MO, USA) in PBS. The injector cannula was left in place for approximately 30 s to allow for diffusion before dummy cannulas were placed back in guide cannulas.

Mice treated peripherally were weighed as described for central LPS studies. Mice were treated IP with either endotoxin-free injectable saline or LPS (830 μg/kg body weight) mixed with injectable saline.

### Locomotor activity

To evaluate the effects of saline or LPS on exploratory locomotor activity, mice were tested 24 h after treatment. Mice were placed in clear plexiglass cages identical to their home cage but devoid of bedding or nesting material. Clear plexiglass lids were placed on top of test cages to prevent escape while facilitating video recording of mice. Locomotor activity was assessed by virtual division of the cage into equal quadrants and then tallying the number of line crossings and rearings each mouse displayed during the 5-minute test period. Videos were scored by trained personnel blinded to treatment.

### Forced swim test

To determine whether LPS injected ICV or IP induced differential depression-like behaviors of ICE KO mice compared to WT mice, we utilized a modified version of the Porsolt Forced Swim Test [32]. Mice were placed in a white plastic container (20 cm diameter × 24 cm tall) that was partially filled with 24 ± 0.5°C water. Test duration was 5 min and the mice were video-recorded for analysis. Videos were scored by trained personnel blinded to treatment. Time of immobility was defined as the time when the mouse’s effort was only that necessary to remain afloat. The forced swim test was administered 24 h after treatment.

### Sucrose preference test

To quantify inflammation-induced anhedonia, which is a common symptom of major depression, we subjected mice to the two-bottle sucrose preference test. This test measures preference for sweetened solution over water. Approximately 1 week prior to treatment, mice were trained by simultaneous presentation with a bottle of water and a bottle of 1% (wt/vol) sucrose solution. Bottles were weighed prior to being placed on the lid of the mouse’s home cage and left in place for 24 h periods. Mice were allowed *ad libitum* access to the bottles. After 24 h, the bottles were reweighed to determine the amount of sucrose solution and water that had been consumed. Preference was calculated as a percentage of sucrose solution consumed compared to the total fluid intake (sucrose/(sucrose + water) * 100). Mice were trained until a stable baseline preference was established and then treatments were administered. Following treatment, sucrose preference testing was conducted 24 to 48 h following treatment. This time frame corresponded with amelioration of overt sickness response and presence of depression-like behavior, as assessed by the FST.

### Tissue collection

At either 4 or 24 h post-injection, mice were euthanized by CO_2_ asphyxiation. Brains were removed and longitudinally cut into hemispheric sections and immediately frozen in sample tubes placed on dry ice. The tissue was stored frozen at −80°C until processing.

### Tissue processing and quantitative real-time RT-PCR (qRT-PCR) analysis

Expression of cytokines and genes associated with immune activation was measured in brain to determine if ICE KO mice had differential pro- or anti-inflammatory responses to LPS compared to WT mice. One hemisphere of each brain was removed from storage and 3 mL of cold Trizol reagent (Invitrogen, Carlsbad, CA, USA) were added to each sample. The tissue was then homogenized using an ultrasonic tissue disruptor (Sonics and Materials Inc., Newborn, CT, USA). An E.Z.N.A kit was used to isolate total RNA (Omega Bio-tek, Norcross, GA, USA). RNA purity (OD 260/280) and quantity was assessed using a Nanodrop Spectrophotometer (Nanodrop Products, Wilmington, DE, USA) and submitted to reverse transcription using a High Capacity cDNA Reverse Transcription kit (Applied Biosystems, Foster City, CA, USA). The cDNA samples were analyzed using qRT-PCR with the Prism 7900HT Fast Real-Time PCR System (Applied Biosystems, Foster City, CA, USA). TaqMan gene expression assays (Applied Biosystems, Foster City, CA, USA) or PrimeTime qPCR assays (Integrated DNA Technologies, Coralville, IA, USA) were used for the detection of IL-1β/pro-IL-1β (catalog no. Mm00434228_m1), IL-1RA (Mm00446186_m1), ICE (Mm.PT.49a.21858521), IL-18 (Mm00434225_m1), TNF-α (Mm00443260_g1), IL-6 (Mm00446190_m1), IL-10 (Mm00439614_m1), CD11b (Mm00434455_m1), MHC II (Mm00439226_m1), GFAP (Mm00546086_m1), and GAPDH (Mm99999915_g1). All assays except for ICE and IL-1R1 were purchased from Applied Biosystems. Samples were analyzed in duplicate using 125 ng of cDNA template mixed with Taqman Universal Master Mix and target primers for each reaction according to the manufacturer’s instructions. Relative quantitative measurement of target gene levels was performed using the ^ΔΔ^Ct method, where Ct is the comparative threshold concentration. GAPDH was used as the endogenous housekeeping control gene to which all other genes were compared.

### Statistical analysis

Data are represented as the means ± SEM. All measures were analyzed using two-way analysis of variance (ANOVA). When the two-way interaction *P* value was <0.05, post-hoc analysis using Fisher’s protected least significant difference test was employed to test for differences among means.

## Results

### Sickness responses were similar in WT and ICE KO mice following central LPS injection

LPS administered ICV decreased body weight of both WT and ICE KO mice (Table 1) over the 24-h period following treatment. Only the main effect of LPS was statistically significant (LPS main effect, F_1,24_ = 45.50, *P* <0.01). Locomotor activity was tested 24 h following treatment (Table 1). LPS, given ICV, did not affect line crossings (LPS main effect, F_1,26_ = 0.28, *P* >0.05) and rearings (LPS main effect, F_1,26_ = 0.002, *P* >0.05) of either strain 24 h after treatment. However, ICE KO mice displayed reduced line crossings (strain main effect, F_1,26_ = 27.50, *P* <0.01) and rearings (strain main effect, F_1,26_ = 21.62, *P* <0.01) compared to WT mice. Despite the baseline difference in activity between the two strains, these responses indicate that deletion of ICE does not change the sickness response when LPS is administered centrally.

**Table 1:** Sickness response measures following ICV LPS

	**Δ Body weight (g / 24 h)**	**Line crossings (# / 5 min)**	**Rearings (# / 5 min)**
WT	−0.10 ± 0.12^a^	62.7 ± 5.5^a^	50.3 ± 5.0^a^
WT-LPS	−1.83 ± 0.39^b^	63.3 ± 6.5^a^	47.9 ± 5.0^a^
ICE KO	0.17 ± 0.13^a^	30.0 ± 2.6^b^	25.7 ± 1.9^b^
ICE KO-LPS	−1.64 ± 0.12^b^	35.5 ± 6.4^b^	28.5 ± 5.2^b^

Body weight was measured at 0 and 24 h and body weight change was calculated from these measures; locomotor activity was tested 24 h after treatment. Averages within columns with different superscript letters are significantly different; *P* <0.05.

### ICE KO mice were protected from central LPS-induced depression-like behavior

**Figure 1:**
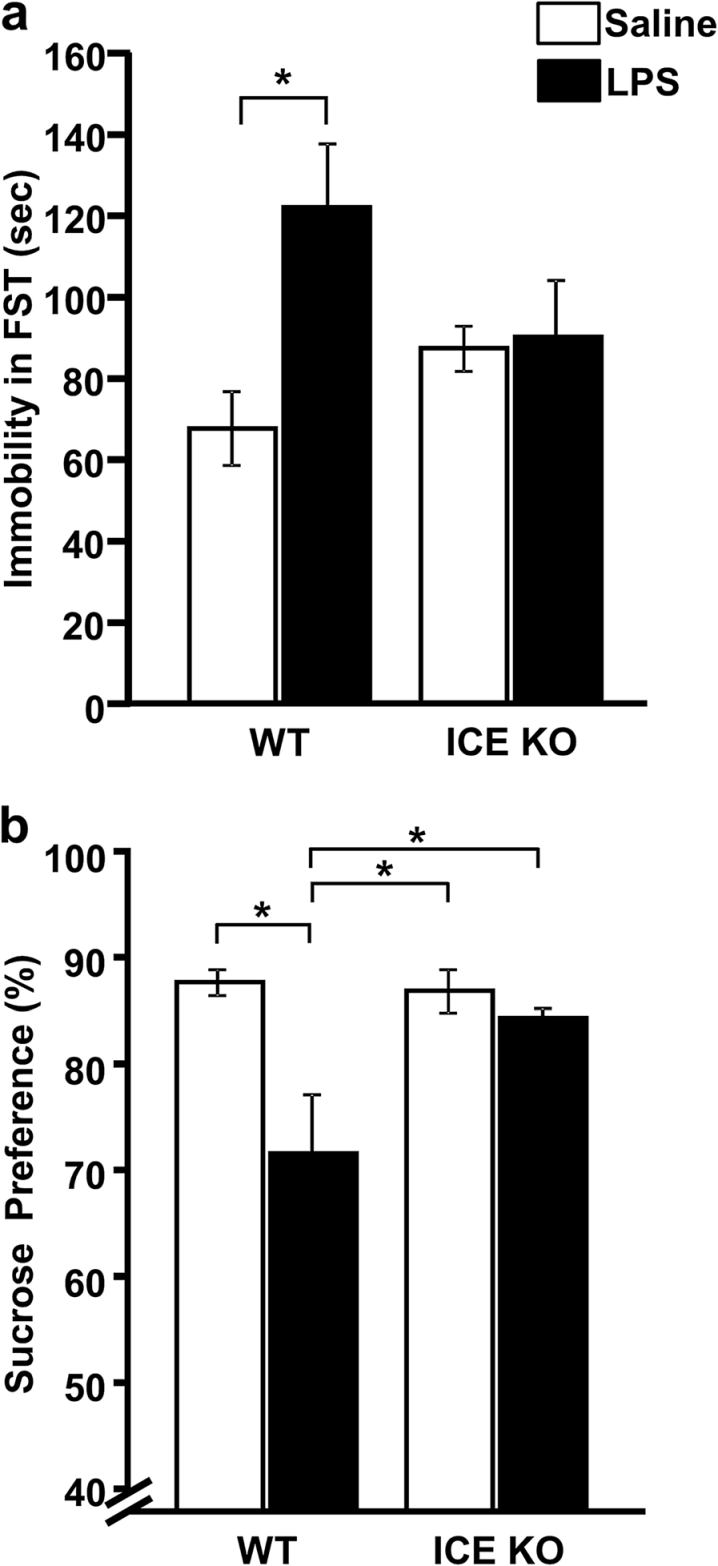
**ICE KO mice were protected from depression-like behaviors following ICV administration of LPS compared to WT mice.** (**a**) Time of immobility in the FST was determined 24 h after treatment. WT mice given LPS had greater time spent immobile compared to control WT mice, while immobility time in LPS-treated ICE KO mice was not different from control mice. (**b**) WT, but not ICE KO, mice displayed decreased preference for sucrose during the 24 to 48 h period following LPS injection. Data represent averages ± SEM. *n* = 9 to 12 mice per group, *P <0.05 comparing bracketed treatment groups.

To test the hypothesis that ICE KO mice would be protected from central LPS-induced depression-like behavior, we employed the FST and sucrose preference test following ICV treatment with LPS or saline. Importantly and in agreement with our hypothesis, LPS increased (strain x LPS interaction; F_3,24_ = 4.35, *P* <0.05) immobility during the FST (Figure 1a) of WT but not ICE KO mice. Similarly, ICE KO but not WT mice maintained (strain x LPS interaction; F_3,40_ = 6.56, *P* <0.05) their preference for a 1% sucrose solution over water following LPS treatment (Figure 1b). Taken together, these data confirm that ICE is necessary for expression of depression-like behavior following central administration of LPS.

### Steady-state expression of brain inflammatory markers declined faster in ICE KO compared to WT mice

To identify underlying substrates in ICE KO mice that are related to them being protected from ICV LPS-induced depression-like behavior, we utilized qRT-PCR to measure expression of cytokines and markers of active glia in brain collected 4 and 24 h after treatment. As expected, LPS induced (*P* <0.01; data not shown) expression of ICE in WT mice at both time points although central LPS did not change IL-18 expression (data not shown).

**Figure 2:**
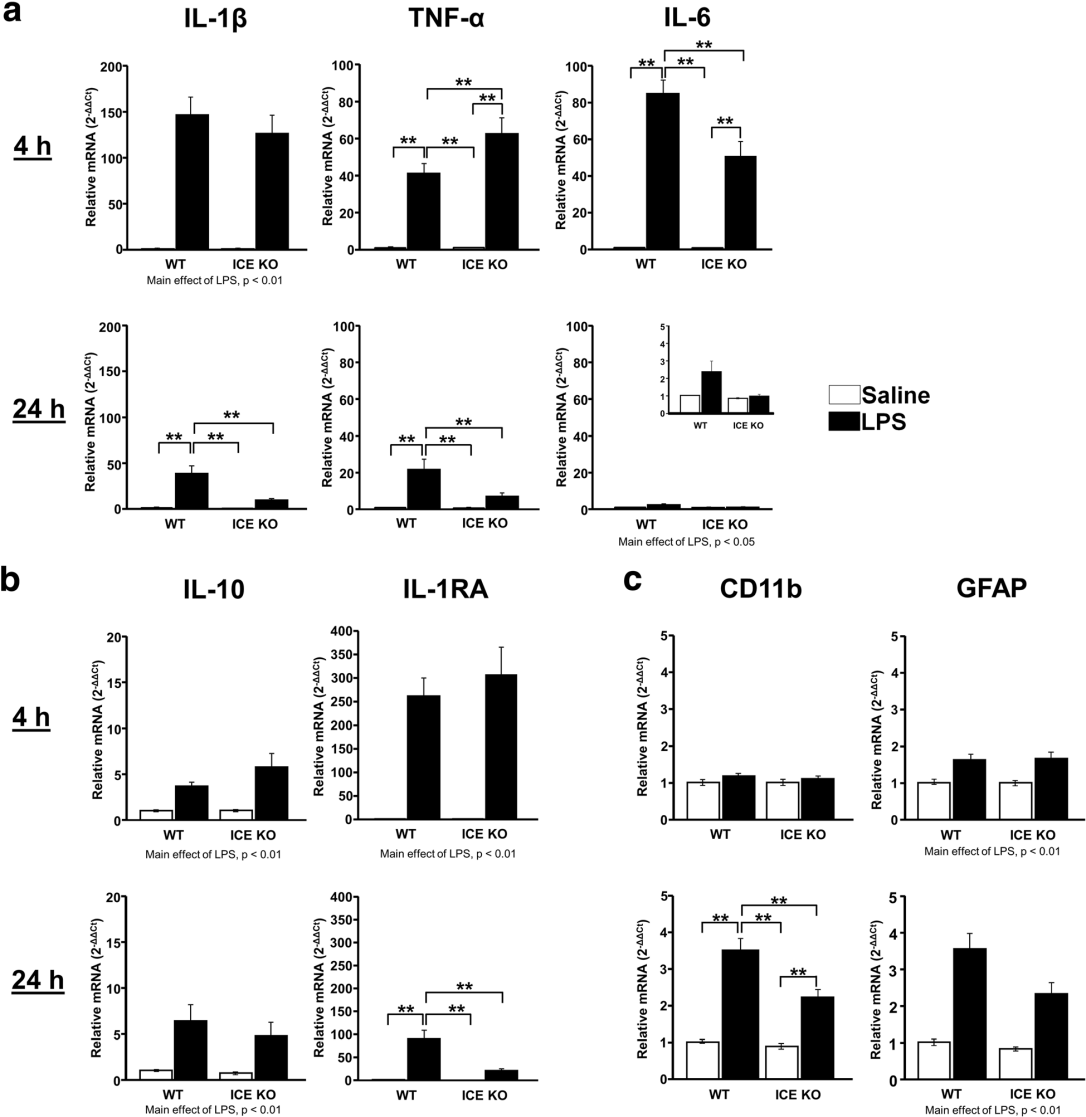
**Expression of pro-inflammatory cytokines in brain was reduced in ICE KO mice at 24 h following LPS given ICV.** (**a**) LPS increased mRNA expression of IL-1β, TNF-α, and IL-6 in WT and ICE KO at 4 h. Expression of IL-1β and TNF-α remained elevated in brains of only WT mice at 24 h. (**b**) IL-10 expression was increased similarly by central LPS in both strains of mice at 4 h and 24 h. In contrast, IL-1RA mRNA expression increased in both strains at 4 h but remained elevated at 24 h only in WT mice. (**c**) CD11b and GFAP expression were increased similarly in WT and ICE KO brains at 4 h. At 24 h, CD11b expression was reduced in ICE KO mice compared to WT mouse brains following ICV administration of LPS. Data are average mRNA expression levels relative to GAPDH ± SEM; **P <0.01, *P <0.05 comparing bracketed groups; main effects of LPS where indicated; *n* = 6 mice per group. Insets: Data presented with zoomed in scale to ease interpretation. Mean Ct values for saline treated WT groups were: IL-1β, 31.7 ± 0.4; TNF-α, 32.0 ± 0.3; IL-6, 31.6 ± 0.4; IL-10, 34.6 ± 0.2; IL-1RA, 33.2 ± 0.3; CD11b, 20.7 ± 0.03; GFAP, 17.4 ± 0.1.

To determine whether ICE deletion reduces neuroinflammation, we examined brain expression of pro-inflammatory cytokines (Figure 2a). LPS increased (LPS main effect; F_1,22_ = 91.63, *P* <0.01) IL-1β expression in both WT and ICE KO mice at 4 h indicating that LPS induces a similar initial inflammatory response in both strains. However, at 24 h, expression of IL-1β remained elevated (strain x LPS interaction; F_3,20_ = 10.09, *P* <0.01) only in WT mice. LPS-treated ICE KO and WT mice had increased (LPS main effect; F_1,22_ = 4.36, *P* <0.01) brain expression of TNF-α mRNA at 4 h compared to controls, but TNF-α expression returned (strain x LPS interaction; F_3,20_ = 5.72, *P* <0.05) to control levels in ICE KO mice by 24 h after treatment while TNF-α expression remained elevated in WT mice. At 4 h after treatment, LPS induced IL-6 expression (strain × LPS interaction; F_3,20_ = 10.19, *P* <0.05) but the LPS response was smaller in ICE KO mice compared to LPS-treated WT mice. At 24 h, brain IL-6 expression was increased by LPS (LPS main effect; F_1,22_ = 5.52, *P* <0.05). However, ICE KO mice tended (strain x LPS interaction; F_3,20_ = 3.84, *P* = 0.06) to have reduced brain IL-6 expression 24 h after ICV injection of LPS.

We examined expression of IL-10 and IL-1RA within the brain to determine if ICE deletion leads to increased expression of anti-inflammatory genes (Figure 2b). IL-10 mRNA increased similarly in both strains at 4 (LPS main effect; F_1,22_ = 22.38, *P* <0.01) and 24 h (LPS main effect; F_1,22_ = 16.00, *P* <0.01) after treatment. IL-1RA, which is an IL-1β-signaling antagonist, was increased (LPS main effect; F_1,22_ = 66.24, *P* <0.01) in both strains in response to central LPS. However, in agreement with IL-1β data, IL-1RA remained elevated (strain x LPS interaction; F_3,20_ = 13.10, *P* <0.01) only in LPS-treated WT mice at 24 h.

Consistent with reductions in pro-inflammatory cytokine expression, the expression of genes associated with glial activation was diminished in LPS-treated ICE KO mice compared to LPS-treated WT mice (Figure 2c). CD11b expression was not influenced by LPS 4 h after treatment but was significantly lower (strain x LPS interaction; F_3,20_ = 8.55, *P* <0.01) in LPS-treated ICE KO mice compared to LPS-treated WT mice at 24 h. LPS increased (LPS main effect; F_1,22_ = 24.65, *P* <0.01) expression of the astrocyte activation marker GFAP at 4 h post treatment in both strains of mice. ICE KO mice tended to have lower expression (strain x LPS interaction; F_1,22_ = 3.87, *P* = 0.06) of GFAP at 24 h.

The similar expression of cytokines in the brains of ICE KO and WT mice at 4 h agrees with their similar sickness response. The reduced cytokine expression in the brains of ICE KO mice compared to WT mice, at 24 h, coincides with the lack of depression-like behaviors following ICV LPS of ICE KO mice, indicating that central cytokines are involved in depression-like behaviors associated with neuroinflammation.

### Systemic LPS administration induced a similar sickness response in ICE KO and WT mice

To determine if sickness responses are similar in ICE KO and WT mice following peripheral administration of LPS, body weight was recorded immediately prior to and 24 h after treatment in both strains of mice (Table 2). Change in body weight was calculated and used as one index of sickness following systemic LPS challenge. Both ICE KO and WT mice treated with LPS displayed reduced (LPS main effect; F_1,45_ = 692.01, *P* <0.01) body weight at 24 h post treatment. ICE KO and WT mice had similar sickness response following IP LPS challenge, indicating that ICE deletion does not prevent sickness response to systemic LPS. Locomotor activity was also tested in all mice 24 h post-treatment (Table 2). WT but not ICE KO mice treated with LPS had a reduced (strain x LPS interaction; F_3,43_ = 10.56, *P* <0.01) number of line crossings and rearing in this locomotor activity test compared to saline treated controls. As observed in the central LPS studies, ICE KO mice, regardless of treatment, had fewer line crossing and rearings compared to saline-treated WT mice (*P* <0.01). ICE KO mice appear to be recovered from reductions in locomotor activity following systemic LPS, although their loss of body weight is similar.

**Table 2:** Sickness response measures following IP LPS

	**Δ Body weight (g / 24 h)**	**Line crossings (# / 5 min)**	**Rearings (# / 5 min)**
WT	−0.02 ± 0.11^a^	60.3 ± 5.6^a^	42.9 ± 5.0^a^
WT-LPS	−2.84 ± 0.09^b^	36.8 ± 6.5^b^	26.9 ± 3.0^b^
ICE KO	−0.09 ± 0.10^a^	35.6 ± 3.8^b^	27.0 ± 3.0^b^
ICE KO-LPS	−2.82 ± 0.12^b^	36.9 ± 2.6^b^	25.2 ± 2.4^b^

Body weight was measured at 0 and 24 h and body weight change was calculated from these measures; locomotor activity was tested 24 h after treatment. Averages within columns with different superscript letters are significantly different; *P* <0.05.

### Peripheral LPS increased FST immobility and decreased sucrose preference of both WT and ICE KO mice

**Figure 3:**
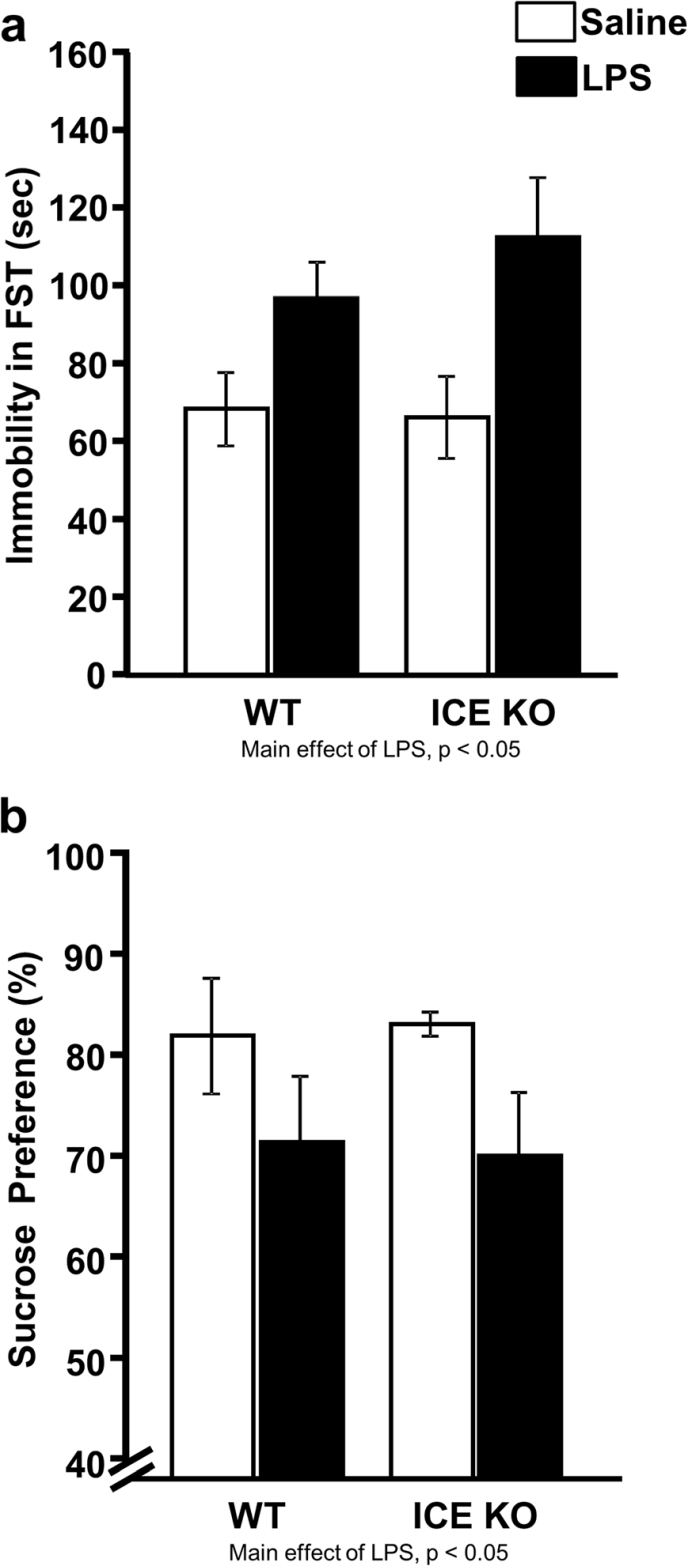
**Both ICE KO and WT mice displayed depression-like behaviors following IP LPS challenge.** LPS given systemically increased (**a**) immobility in the FST 24 h after treatment and decreased (**b**) sucrose preference when measured 24 to 48 h post-treatment similarly in both mouse strains. Data represent averages ± SEM; main effects of LPS where indicated; *n* = 6 mice per group.

To test the hypothesis that ICE KO mice would not be protected from systemic LPS-induced depression-like behavior, we submitted both strains of mice to FST and sucrose preference test following IP administration of LPS or saline. During the FST, LPS increased (LPS main effect; F_1,45_ = 10.33, *P* <0.01) time of immobility similarly of both WT and ICE KO mice (Figure 3a). To determine whether ICE KO mice developed an anhedonic response, preference for a 1% sucrose solution from 24 to 48 h post treatment was quantified (Figure 3b). Both WT and ICE KO mice treated IP with LPS had reduced (LPS main effect; F_1,22_ = 4.76, *P* <0.05) sucrose preference. These data confirm our hypothesis that deletion of ICE does not protect against depression-like behavior following systemic administration of LPS.

### A peripheral LPS challenge increased expression of pro-inflammatory cytokines and glial activation markers similarly in the brains of both WT and ICE KO mice

**Figure 4:**
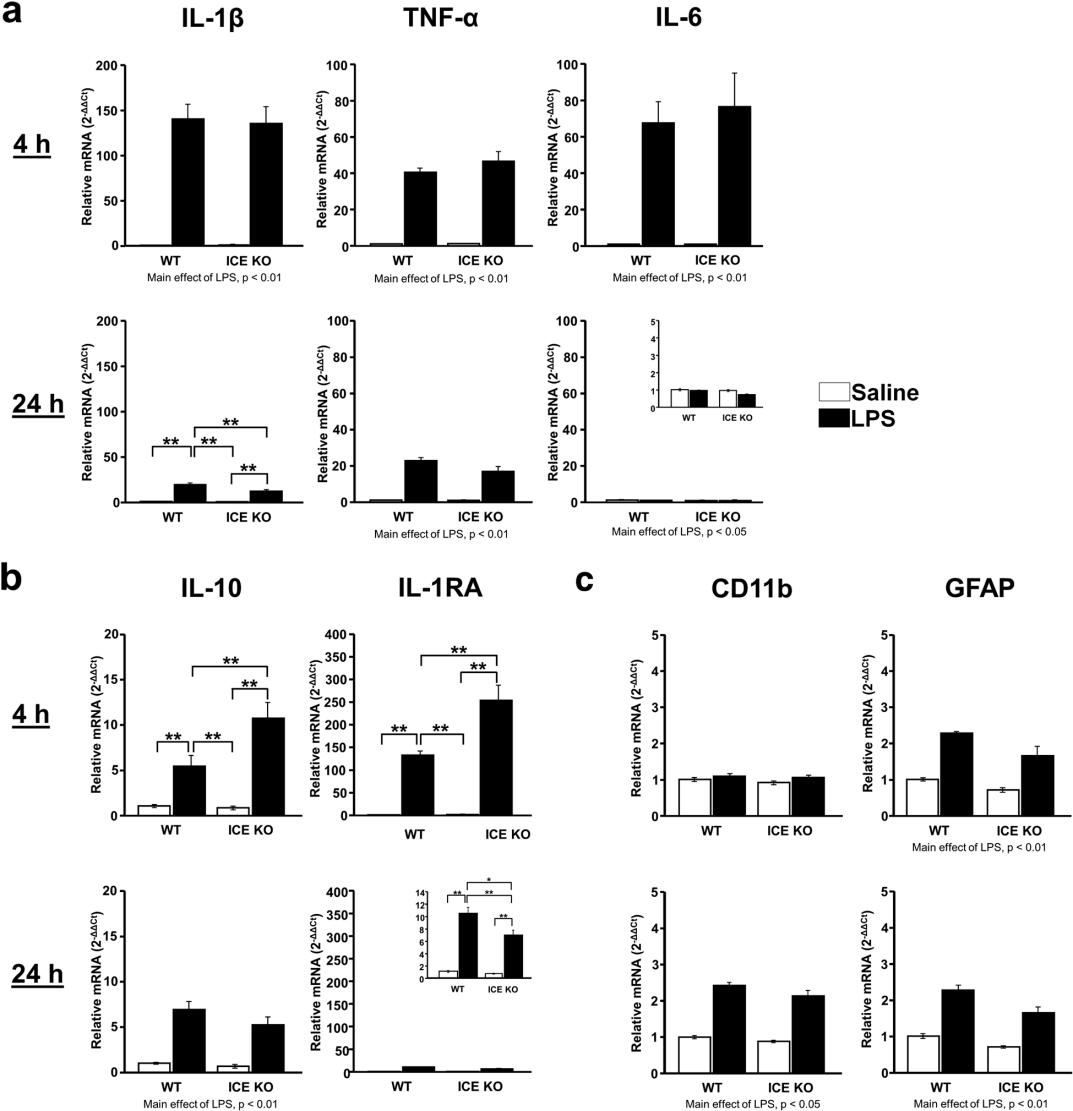
**Both ICE KO and WT mice given systemic LPS exhibited increased expression of inflammatory markers in brain.** In both WT and ICE KO mice, LPS injected IP induced expression of (**a**) IL-1β, TNF-α, and IL-6 at 4 h. All but IL-6 remained elevated at 24 h. (**b**) ICE KO mice had greater expression of IL-10 and IL-1RA at 4 h. However, at 24 h, the LPS-induced increase in IL-10 expression was similar in both strains of mice while that of IL-1RA was reduced in ICE KO mice. (**c**) CD11b was not elevated by LPS until 24 h post-treatment while GFAP expression was increased at both 4 h and 24 h similarly in WT and ICE KO mice. Data are average mRNA expression levels relative to GAPDH ± SEM, **P <0.01, *P <0.05 comparing bracketed groups; main effects of LPS where indicated; *n* = 5 to 6 mice per group. Insets: Data presented with zoomed in scale to ease interpretation. Mean Ct values for saline treated WT groups were: IL-1β, 29.5 ± 0.1; TNF-α, 29.6 ± 0.1; IL-6, 29.2 ± 0.1; IL-10, 34.9 ±0.1; IL-1RA, 30.4 ± 0.3; CD11b, 21.6 ± 0.1; GFAP, 18.0 ± 0.1.

To evaluate whether LPS increased steady-state expression of mRNA for inflammatory mediators similarly in WT and ICE KO mice, qRT-PCR was used to quantify mRNA expression in brain after IP treatment with LPS or saline. Consistent with data from the central LPS studies, ICE expression was increased (*P* <0.05) at 4 and 24 h by LPS in WT mice only. Interestingly, systemic LPS increased (LPS main effect; F_1,21_ = 5.53, *P* <0.05) brain IL-18 expression in both strains at 4 h but not at 24 h (*P* >0.1; data not shown). Systemic LPS (Figure 4a) similarly increased (LPS main effect; F_1,21_ = 127.96, *P* <0.01) brain expression of IL-1β in both WT and ICE KO mice at 4 h after treatment. However at 24 h, ICE KO mice had lower (strain x LPS interaction; F_3,19_ = 6.58, *P* <0.05) IL-1β expression relative to LPS-treated WT mice 24 h after treatment. In contrast to central LPS-treated ICE KO mice, IL-1β remained elevated in systemic LPS-treated ICE KO mice relative to controls. Systemic LPS increased brain TNF-α (LPS main effect; F_1,21_ = 192.89, *P* <0.01) and IL-6 (LPS main effect; F_1,21_ = 41.72, *P* <0.01) expression at 4 h. TNF-α expression remained elevated (LPS F_1,21_ = 121.70, *P* <0.01) at 24 h, while IL-6 was reduced (LPS main effect; F_1,21_ = 5.68, *P* <0.05) in both strains of mice. Importantly, there were no strain differences in TNF-α or IL-6 expression.

Brain expression of IL-10 and IL-1RA was measured to determine whether these transcripts were similar in ICE KO and WT mice treated with systemic LPS (Figure 4b). Four hours following an IP injection of LPS, IL-10 (strain x LPS interaction; F_3,19_ = 6.83, *P* <0.05) and IL-1RA (strain x LPS interaction; F_3,19_ = 6.83, *P* <0.05) expression were greater in ICE KO mice compared to LPS-treated WT mice. By 24 h, both strains had similar increases (LPS main effect; F_1,21_ = 68.83, *P* <0.01) in brain IL-10. However, LPS-induced IL-1RA expression was lower (strain × LPS interaction; F_3,19_ = 7.34, *P* <0.05) in ICE KO than WT mice at 24 h after treatment but still remained elevated compared to control mice.

To determine if ICE influenced activation of glia in response to systemic LPS, we measured expression of microglia and astrocyte activation markers in brain (Figure 4c). LPS induced CD11b (LPS main effect; F_1,21_ = 200.97, *P* <0.01) expression similarly in both ICE KO and WT mice at 24 h only. Brain GFAP expression was increased similarly at 4 h (LPS main effect; F_1,21_ = 9.06, *P* <0.01) and 24 h (LPS main effect; F_1,21_ = 96.48, *P* <0.01)in both strains following peripheral LPS treatment. These data indicate that glial activation is similar in both ICE KO and WT following systemic LPS, unlike what was observed in ICE KO mice following central injection of LPS.

With the exception of IL-1β and IL-1RA expression, both ICE KO and WT mice have similar expression of genes in the brain that are associated with inflammation following systemic LPS. These gene expression data agree well with the induction of a similar depression-like behavior in both strains following systemic LPS.

## Discussion

We previously demonstrated that ICE KO mice are resistant to central LPS-induced reduction in food intake [24] and feeding behavior [23]. However, ICE KO mice lack this protection following systemic LPS administration [23] although they are protected against peripheral endotoxic shock induced by higher doses of LPS [33]. These findings suggested that there is a distinction between responses elicited by an activation of the central innate immune system versus the peripheral immune system. Given the more recent data showing that IL-1 is critically involved in the development of depression-like behaviors [10,16,34,35], these findings stimulated us to test the hypothesis that ICE KO mice might be protected from central but not peripheral LPS-induced depression-like behaviors. To our knowledge, we are now the first to report that ICE is required for development of depression-like behavior following a central, but not systemic, LPS challenge.

ICE is a cysteine protease and is the enzyme principally responsible for cleavage of two critical pro-inflammatory cytokines, pro-IL-1β and pro-IL18, from their inactive precursors to their mature active secreted forms. ICE is constitutively present within cells as an inactive precursor co-localizing with a group of proteins that collectively form the inflammasome. Inflammasomes contain nucleotide and oligomerization domain-like receptor (NLR) family proteins that act as intracellular receptors for pathogen-associated molecular patterns (PAMPs) or danger-associated molecular patterns (DAMPs) in a similar fashion as the widely recognized toll-like receptors [22,36,37]. Following recognition of PAMPs and DAMPs by NLRs, there is a transient increase in ICE activity [20]. The precise mechanism by which LPS can directly activate inflammasomes and ICE activity has not been fully elucidated. However, LPS-induced upregulation of ICE expression has largely been shown to occur in cells of the myeloid lineage such as central microglia [18] and peripheral monocytes/macrophages [19]. We confirmed that ICE expression in the brain was upregulated by both central and peripheral LPS in WT mice.

ICE activity has substantial influence over behavioral responses during inflammation, presumably through its role in processing inactive IL-1β [20]. Genetic deletion of ICE decreases LPS-induced IL-1β secretion [33]. It has been well established that IL-1β activates the hypothalamic-pituitary-adrenal axis and induces behavioral changes associated with sickness and depression, such as anhedonia, disruption of sleep, cognitive disturbances, temperature disregulation, and consumption of food [9,26,27]. Our findings indicate a prominent role for central ICE during inflammation-induced depression. These new data are in in agreement with recent work that has demonstrated a role for IL-1ß in the development of depression-like behavior utilizing a variety of models. Chronic exposure to IL-1β diminished sucrose preference and social exploration; which are indicative of the depressive symptoms of anhedonia and social withdrawal, respectively [10,28]. Chronic mild stress (CMS) of mice not only elevates IL-1β levels but also results in depression-like symptoms, including a decrease in sucrose preference (to the point of aversion) and decreased social exploration. These symptoms are dependent on IL-1β as either type 1 IL-1 receptor (IL-1R) deficiency or the injection of IL-1 receptor antagonist (IL-1RA) block the effect of CMS [10]. Similarly, several other studies using models of CMS, chronic unpredictable stress (CUS), chronic pain, and ischemic stroke have demonstrated that reducing IL-1 signaling blocks depression-like behaviors including reduced sucrose preference and increased immobility time in the FST [16,34,35]. Similar CUS causes mice to display decreased preference for sucrose solution compared to unstressed mice, and this anhedonic response is blocked by ICV administration of IL-1RA [34]. This later finding is important as it illustrates that IL-1β activity within the brain is required for the development of depression-like behavior. Chronic pain has also been linked to depression with a possible cause and effect relationship [38,39]. Moreover, IL-1 has been extensively examined for its role in mediating symptoms of neuropathic pain (reviewed in [40]). Utilizing the spared nerve injury model of chronic pain, increased time of immobility during the FST was blocked by central administration of IL-1RA into the lateral ventricle. Middle cerebral artery occlusion, which is a model of ischemic stroke, leads to a robust induction of brain IL-1β expression and IL-1 signaling in this model plays an important role in post-stroke depression as evidenced by a reduction in sucrose consumption that is blocked when mice are treated with an ICV injection of IL-1RA [35]. All of these studies established that brain IL-1 signaling is of significant importance for mediating depression-like behaviors since each model targeted IL-1 signaling in the brain. All of these results are in agreement with the new findings reported here that deletion of ICE blocks the LPS-induced increase in FST immobility and decrease in sucrose preference only when LPS is administered centrally. Clearly, non-ICE dependent mechanisms for depression-like behavior remain functional when LPS is administered peripherally.

Despite reduced IL-1β secretion, ICE KO mice have similar a sickness response compared to WT mice in terms of loss of body weight following both systemic and central LPS challenges. This lack of attenuation of the sickness response is not surprising since IL-1R1 knockout mice also lose body weight similar to WT mice when treated with ICV or IP LPS [41]. Further, inhibiting IL-1 signaling with ICV or IP administration of IL-1RA is not sufficient to block body weight loss in LPS treated rats [42]. It was postulated several years ago that LPS-induced sickness behaviors require the presence of either TNF-α or IL-1β, but when IL-1 signaling is absent, TNF-α assumes a more prominent role [41]. This postulate is consistent with the present findings that both WT and ICE KO mice displayed elevated TNF-α expression in the brain following LPS. Therefore, similar degrees of sickness behavior following LPS injection in WT and ICE KO mice is consistent with the known pleiotropic properties of both IL-1β and TNF-α.

Unexpectedly, ICE KO mice had reduced locomotor activity as assessed by the number of line crossings and rearings compared to WT mice. A possible explanation for reduced exploratory behavior measured in ICE KO mice is increased anxiety in these mice. Exploratory behavior is often used as an important screening tool for anxiety-like behavior [43]. We cannot rule out the possibility that reduced locomotor activity exhibited by ICE KO mice is indicative of an anxiogenic phenotype but our experiments were not designed to properly test this possibility. However, it is important to note that locomotor activity of ICE KO mice 24 h after LPS was unaffected by either central or systemic LPS challenge. We interpret these data to suggest that ICE KO mice display a more rapid recovery from sickness behavior compared to WT mice. Again, testing this possibility was not the focus of our experiments. Importantly for the current body of work, the decrease in locomotor activity did not translate into an elevated time of immobility during the FST. The equal time of immobility of saline-treated WT and ICE KO mice during this test indicates that reduced locomotor activity was not due to depression-like behavior and that differences in performance during the FST did not result from a motor deficit. However, as ICE inhibitors may draw increasing interest as treatments for inflammation-associated diseases, it will be important to evaluate different alterations for other ICE-dependent behaviors.

Cytokine signals are propagated diffusely throughout the brain following an initial immune stimulation. This propagation occurs primarily by the induction of additional IL-1ß and other pro-inflammatory cytokines in brain resident cells including microglia [44-48]. Despite having reduced IL-1β secretion in response to LPS [33], ICE KO mice still develop depression-like behavior following systemic but not central LPS administration. This finding alludes to compensatory actions of other peripheral cytokines to induce inflammatory mediators within brain despite the deficiency in mature IL-1β, or a lack of peripheral IL-1ß involvement in peripherally induced depression-like behaviors. In our experiments, both WT and ICE KO mice displayed increased early (4 h) expression of brain IL-1β, TNF-α and IL-6 following both systemic and central LPS. At 24 h, brain IL-1β and TNF-α were no longer elevated in central LPS-treated ICE KO compared to saline-treated ICE KO mice even though expression of these genes remained elevated in LPS-treated WT mice. In our model, the reduced induction of IL-1β mRNA expression following LPS treatment likely results from a quicker extinguishing of the feed-forward cytokine signaling, in agreement with data demonstrating that IL-1β induces its own expression and the expression of TNF-α and IL-6 within brain [49,50]. This was in contrast to what was observed following systemic LPS because most inflammatory mediators that we measured were increased similarly in WT and ICE KO mice. In contrast, TNF-α expression remained elevated at 24 h following systemic LPS administration in both WT and ICE KO mice. This finding indicates that even in the absence of IL-1ß secretion, TNF-α may mediate depression-like behavior because its absence in the brain at 24 h in ICV-treated ICE KO mice corresponds to a lack of depression-like behavior and its continued presence at 24 h in ICV-LPS WT, IP-LPS WT, and IP-LPS ICE KO mice corresponds to the presence of depression-like behaviors. A role for TNF-α in depression-like behavior has been directly shown. Even extremely low doses of TNF-α administered ICV causes depression-like behavior as assessed as increased time of immobility during both the FST and tail suspension test [51]. In addition, TNF-R1 deficient mice and mice treated with a neutralizing antibody to TNF-α had a decreased time of immobility during the FST, indicating an anti-depressant response. This study supports work showing that TNF receptor deficient mice have lower immobility during the FST, again indicating an anti-depressant response. The TNF receptor deficient mice also have increased consumption of a sucrose solution, indicative of an anhedonic response mediated by TNF-α [52]. In further support of a role for TNF-α in depression, human patients afflicted with plaque psoriasis showed significant improvement in Beck Depression Inventory and Hamilton Rating Score for depression when treated with the TNF neutralizing drug Etanercept [53]. Patients treated with Etanercept showed significant improvement in sexual function, sleep, irritability, and other symptoms of depression that impacted quality of life compared to patients receiving placebo. These data indicate that TNF-α is probably involved in mediating depression-like behaviors. Together with our current data, we hypothesize that in the absence of IL-1ß, depression-like behavior is present only when central TNF-α expression is elevated following the LPS challenge.

Expression of IL-10 and IL-1RA in the brain remain elevated in ICE KO and WT mice following systemic LPS exposure at 24 h. This was not the case following central LPS, further supporting a role for IL-1β in a sustained brain inflammatory response. Compensatory actions of other cytokines such as TNF-α [54] contribute to peripheral immune activation cascades when IL-1 action is lost. We also found that ICE KO mice have decreased mRNA expression of genes associated with microglia and astrocyte activation, CD11b and GFAP, following central LPS treatment. We interpret these findings as evidence that IL-1β is important for maintaining activation of glial cells in response to neuroinflammation. Indeed, reduced cytokine expression observed in ICE KO following central LPS is reflective of reduced glial cell activation. We are intrigued by our finding that brain MHCII expression was not different between ICE KO and WT mice following central LPS (data not shown) as this may indicate a less prominent role for IL-1β to induce an antigen presenting phenotype in microglia. Based on these data, our results add to evidence that ICE and subsequently IL-1ß signaling plays a necessary role for initiating and sustaining a full inflammatory response within the brain that manifests behaviors associated with depression.

## Conclusions

To our knowledge, we are first to report that ICE KO mice are protected from central LPS-induced depression-like behavior. Deletion of ICE has a significant impact on the inflammatory profile in brain following ICV LPS but essentially no effect on the brain following a peripheral LPS challenge. We propose that targeting of ICE represents a potential therapeutic target directed at treating neuroinflammation-dependent comorbid depression.

## Abbreviations

CD-11b: Cluster of differentiation molecule 11b; CMS: Chronic mild stress; CUS: Chronic unpredictable stress; DAMP: Damage associated molecular pattern; FST: Forced swim test; GFAP: Glial fibrillary acidic protein; ICE: Interleukin-1 Beta converting enzyme; ICV: Intracerebroventricular; IL-18: Interleukin 18; IL-1β: Interleukin-1 beta; IL-1RA: Interleukin-1 receptor antagonist; IL-6: Interleukin 6; IL-10: Interleukin 10; IP: Intraperitoneal; KO: Knockout; LPS: Lipopolysaccharide; MHCII: Major histocompatibility complex 2; NLR: Nucleotide oligomerization domain receptor; PAMP: Pathogen associated molecular pattern; qRT-PCR: Quantitative real-time reverse transcription polymerase chain reaction; TNF-α: Tumor necrosis factor alpha; WT: Wild type.

## Competing interests

All authors declare that they have no conflicts of interest for any data presented in this paper. Keith W. Kelley has received an honorarium from Astra-Zeneca.

## Authors’ contributions

MAL conceived of the study, formulated the design of the studies, carried out the execution and analysis of these studies, and drafted the manuscript. RHM participated in formulating the design of the studies and interpretation of results and helped to draft the manuscript. KWK participated in formulating the design of the studies and interpretation of results and helped to draft the manuscript. All authors have read and approved the final version of the manuscript.

## References

[B1] Smith RS The macrophage theory of depression Medical hypotheses 1991 35 298 306 10.1016/0306-9877(91)90272-Z 1943879

[B2] Beumer W Gibney SM Drexhage RC Pont-Lezica L Doorduin J Klein HC Steiner J Connor TJ Harkin A Versnel MA Drexhage HA The immune theory of psychiatric diseases: a key role for activated microglia and circulating monocytes J Leukoc Biol 2012 92 959 975 10.1189/jlb.0212100 22875882

[B3] Raison CL Miller AH Is depression an inflammatory disorder? Curr Psychiatry Rep 2011 13 467 475 10.1007/s11920-011-0232-0 21927805 PMC3285451

[B4] Bodenheimer T Chen E Bennett HD Confronting the growing burden of chronic disease: can the U.S. health care workforce do the job? Health Aff 2009 28 64 74 10.1377/hlthaff.28.1.64 19124856

[B5] Moussavi S Chatterji S Verdes E Tandon A Patel V Ustun B Depression, chronic diseases, and decrements in health: results from the World Health Surveys Lancet 2007 370 851 858 10.1016/S0140-6736(07)61415-9 17826170

[B6] Hindle JV Ageing, neurodegeneration and Parkinson’s disease Age Ageing 2010 39 156 161 10.1093/ageing/afp223 20051606

[B7] Brookmeyer R Johnson E Ziegler-Graham K Arrighi HM Forecasting the global burden of Alzheimer’s disease Alzheimers Dement 2007 3 186 191 10.1016/j.jalz.2007.04.381 19595937

[B8] Catena-Dell’Osso M Bellantuono C Consoli G Baroni S Rotella F Marazziti D Inflammatory and neurodegenerative pathways in depression: a new avenue for antidepressant development? Curr Med Chem 2011 18 245 255 10.2174/092986711794088353 21110802

[B9] Rothwell NJ Luheshi GN Interleukin 1 in the brain: biology, pathology and therapeutic target Trends Neurosci 2000 23 618 625 10.1016/S0166-2236(00)01661-1 11137152

[B10] Goshen I Kreisel T Ben-Menachem-Zidon O Licht T Weidenfeld J Ben-Hur T Yirmiya R Brain interleukin-1 mediates chronic stress-induced depression in mice via adrenocortical activation and hippocampal neurogenesis suppression Mol Psychiatry 2008 13 717 728 10.1038/sj.mp.4002055 17700577

[B11] O’Connor JC Lawson MA Andre C Moreau M Lestage J Castanon N Kelley KW Dantzer R Lipopolysaccharide-induced depressive-like behavior is mediated by indoleamine 2,3-dioxygenase activation in mice Mol Psychiatry 2009 14 511 522 10.1038/sj.mp.4002148 18195714 PMC2683474

[B12] O’Connor JC Andre C Wang Y Lawson MA Szegedi SS Lestage J Castanon N Kelley KW Dantzer R Interferon-gamma and tumor necrosis factor-alpha mediate the upregulation of indoleamine 2,3-dioxygenase and the induction of depressive-like behavior in mice in response to bacillus Calmette-Guerin J Neurosci 2009 29 4200 4209 10.1523/JNEUROSCI.5032-08.2009 19339614 PMC2835569

[B13] Maes M Song C Yirmiya R Targeting IL-1 in depression Expert Opin Ther Targets 2012 16 1097 1112 10.1517/14728222.2012.718331 22925041

[B14] Miller AH Maletic V Raison CL Inflammation and its discontents: the role of cytokines in the pathophysiology of major depression Biol Psychiatry 2009 65 732 741 10.1016/j.biopsych.2008.11.029 19150053 PMC2680424

[B15] Dinarello CA Interleukin-1 in the pathogenesis and treatment of inflammatory diseases Blood 2011 117 3720 3732 10.1182/blood-2010-07-273417 21304099 PMC3083294

[B16] Norman GJ Karelina K Zhang N Walton JC Morris JS Devries AC Stress and IL-1beta contribute to the development of depressive-like behavior following peripheral nerve injury Mol Psychiatry 2010 15 404 414 10.1038/mp.2009.91 19773812 PMC5214062

[B17] Levine J Barak Y Chengappa KN Rapoport A Rebey M Barak V Cerebrospinal cytokine levels in patients with acute depression Neuropsychobiology 1999 40 171 176 10.1159/000026615 10559698

[B18] Yao J Johnson RW Induction of interleukin-1 beta-converting enzyme (ICE) in murine microglia by lipopolysaccharide Brain Res Mol Brain Res 1997 51 170 178 10.1016/S0169-328X(97)00235-0 9427519

[B19] Li P Allen H Banerjee S Seshadri T Characterization of mice deficient in interleukin-1 beta converting enzyme J Cell Biochem 1997 64 27 32 10.1002/(SICI)1097-4644(199701)64:1<27::AID-JCB5>3.0.CO;2-1 9015751

[B20] Franchi L Eigenbrod T Munoz-Planillo R Nunez G The inflammasome: a caspase-1-activation platform that regulates immune responses and disease pathogenesis Nat Immunol 2009 10 241 247 19221555 10.1038/ni.1703 PMC2820724

[B21] Maslanik T Mahaffey L Tannura K Beninson L Greenwood BN Fleshner M The inflammasome and Danger Associated Molecular Patterns (DAMPs) are implicated in cytokine and chemokine responses following stressor exposure Brain Behav Immun 2012 28 54 62 23103443 10.1016/j.bbi.2012.10.014

[B22] Fleshner M Stress-evoked sterile inflammation, danger associated molecular patterns (DAMPs), microbial associated molecular patterns (MAMPs) and the inflammasome Brain Behav Immun 2013 27 1 7 22964544 10.1016/j.bbi.2012.08.012

[B23] Burgess W Gheusi G Yao J Johnson RW Dantzer R Kelley KW Interleukin-1beta-converting enzyme-deficient mice resist central but not systemic endotoxin-induced anorexia Am J Physiol 1998 274 R1829 R1833 9841556 10.1152/ajpregu.1998.274.6.R1829

[B24] Yao JH Ye SM Burgess W Zachary JF Kelley KW Johnson RW Mice deficient in interleukin-1beta converting enzyme resist anorexia induced by central lipopolysaccharide Am J Physiol 1999 277 R1435 R1443 10564217 10.1152/ajpregu.1999.277.5.R1435

[B25] Besedovsky HO del Rey A Interactions between immunological cells and the hypothalamus pituitary-adrenal axis: an example of neuroendocrine immunoregulation Recenti Prog Med 1988 79 300 304 3264411

[B26] Besedovsky HO del Rey A The cytokine-HPA axis feed-back circuit Z Rheumatol 2000 Suppl 2 II/26 II/30 10.1007/s003930070014 11155800

[B27] Goshen I Yirmiya R Interleukin-1 (IL-1): a central regulator of stress responses Front Neuroendocrinol 2009 30 30 45 10.1016/j.yfrne.2008.10.001 19017533

[B28] Nestler EJ Hyman SE Animal models of neuropsychiatric disorders Nat Neurosci 2010 13 1161 1169 10.1038/nn.2647 20877280 PMC3750731

[B29] Kuida K Lippke JA Ku G Harding MW Livingston DJ Su MS Flavell RA Altered cytokine export and apoptosis in mice deficient in interleukin-1 beta converting enzyme Science 1995 267 2000 2003 10.1126/science.7535475 7535475

[B30] Lawson MA Kelley KW Dantzer R Intracerebroventricular administration of HIV-1 Tat induces brain cytokine and indoleamine 2,3-dioxygenase expression: a possible mechanism for AIDS comorbid depression Brain Behav Immun 2011 25 1569 1575 10.1016/j.bbi.2011.05.006 21620953 PMC3191256

[B31] Fanklin GPKBJ The Mouse Brain in Stereotaxic Coordinates 2001 2 Waltham, MA: Academic

[B32] Porsolt RD Brossard G Hautbois C Roux S Crawley JN Rodent models of depression: forced swimming and tail suspension behavioral despair tests in rats and mice Curr Protoc Neurosci Chapter 8:Unit 8 10A 2001 Hoboken, NJ: John Wiley and Sons 10.1002/0471142301.ns0810as14 18428536

[B33] Li P Allen H Banerjee S Franklin S Herzog L Johnston C McDowell J Paskind M Rodman L Salfeld J Mice deficient in IL-1 beta-converting enzyme are defective in production of mature IL-1 beta and resistant to endotoxic shock Cell 1995 80 401 411 10.1016/0092-8674(95)90490-5 7859282

[B34] Koo JW Duman RS IL-1beta is an essential mediator of the antineurogenic and anhedonic effects of stress Proc Natl Acad Sci U S A 2008 105 751 756 10.1073/pnas.0708092105 18178625 PMC2206608

[B35] Craft TK DeVries AC Role of IL-1 in poststroke depressive-like behavior in mice Biol Psychiatry 2006 60 812 818 10.1016/j.biopsych.2006.03.011 16730336

[B36] Lamkanfi M Dixit VM The inflammasomes PLoS Pathog 2009 5 e1000510 10.1371/journal.ppat.1000510 20041168 PMC2791419

[B37] Iwata M Ota KT Duman RS The inflammasome: pathways linking psychological stress, depression, and systemic illnesses Brain Behav Immun 2013 http://dx.doi.org/10.1016/j.bbi.2012.12.008. [Epub ahead of print] 10.1016/j.bbi.2012.12.008 PMC4426992 23261775

[B38] Fishbain DA Cutler R Rosomoff HL Rosomoff RS Chronic pain-associated depression: antecedent or consequence of chronic pain? A review Clin J Pain 1997 13 116 137 10.1097/00002508-199706000-00006 9186019

[B39] Lepine JP Briley M The epidemiology of pain in depression Hum Psychopharmacol 2004 Suppl 1 S3 S7 15378670 10.1002/hup.618

[B40] Marchand F Perretti M McMahon SB Role of the immune system in chronic pain Nat Rev Neurosci 2005 6 521 532 15995723 10.1038/nrn1700

[B41] Bluthe RM Laye S Michaud B Combe C Dantzer R Parnet P Role of interleukin-1beta and tumour necrosis factor-alpha in lipopolysaccharide-induced sickness behaviour: a study with interleukin-1 type I receptor-deficient mice Eur J Neurosci 2000 12 4447 4456 11122355

[B42] Bluthe RM Dantzer R Kelley KW Effects of interleukin-1 receptor antagonist on the behavioral effects of lipopolysaccharide in rat Brain Res 1992 573 318 320 10.1016/0006-8993(92)90779-9 1387028

[B43] Crawley JN Exploratory behavior models of anxiety in mice Neurosci Biobehav Rev 1985 9 37 44 10.1016/0149-7634(85)90030-2 2858080

[B44] Vitkovic L Konsman JP Bockaert J Dantzer R Homburger V Jacque C Cytokine signals propagate through the brain Mol Psychiatry 2000 5 604 615 10.1038/sj.mp.4000813 11126391

[B45] Quan N Stern EL Whiteside MB Herkenham M Induction of pro-inflammatory cytokine mRNAs in the brain after peripheral injection of subseptic doses of lipopolysaccharide in the rat J Neuroimmunol 1999 93 72 80 10.1016/S0165-5728(98)00193-3 10378870

[B46] Quan N Sundar SK Weiss JM Induction of interleukin-1 in various brain regions after peripheral and central injections of lipopolysaccharide J Neuroimmunol 1994 49 125 134 10.1016/0165-5728(94)90188-0 8294551

[B47] Stern EL Quan N Proescholdt MG Herkenham M Spatiotemporal induction patterns of cytokine and related immune signal molecule mRNAs in response to intrastriatal injection of lipopolysaccharide J Neuroimmunol 2000 109 245 260 10.1016/S0165-5728(00)00318-0 10996227

[B48] Dantzer R Konsman JP Bluthe RM Kelley KW Neural and humoral pathways of communication from the immune system to the brain: parallel or convergent? Auton Neurosci 2000 85 60 65 10.1016/S1566-0702(00)00220-4 11189027

[B49] Taishi P Churchill L De A Obal F Jr Krueger JM Cytokine mRNA induction by interleukin-1beta or tumor necrosis factor alpha in vitro and in vivo Brain Res 2008 1226 89 98 18620339 10.1016/j.brainres.2008.05.067 PMC2642478

[B50] Depino A Ferrari C Pott Godoy MC Tarelli R Pitossi FJ Differential effects of interleukin-1beta on neurotoxicity, cytokine induction and glial reaction in specific brain regions J Neuroimmunol 2005 168 96 110 10.1016/j.jneuroim.2005.07.009 16112750

[B51] Kaster MP Gadotti VM Calixto JB Santos AR Rodrigues AL Depressive-like behavior induced by tumor necrosis factor-alpha in mice Neuropharmacology 2012 62 419 426 10.1016/j.neuropharm.2011.08.018 21867719

[B52] Simen BB Duman CH Simen AA Duman RS TNFalpha signaling in depression and anxiety: behavioral consequences of individual receptor targeting Biol Psychiatry 2006 59 775 785 10.1016/j.biopsych.2005.10.013 16458261

[B53] Tyring S Gottlieb A Papp K Gordon K Leonardi C Wang A Lalla D Woolley M Jahreis A Zitnik R Etanercept and clinical outcomes, fatigue, and depression in psoriasis: double-blind placebo-controlled randomised phase III trial Lancet 2006 367 29 35 10.1016/S0140-6736(05)67763-X 16399150

[B54] McCusker RH Kelley KW Immune-neural connections: how the immune system’s response to infectious agents influences behavior J Exp Biol 2013 216 84 98 10.1242/jeb.073411 23225871 PMC3515033

